# Comparison of Ultrasound Attenuation Imaging Using a Linear versus a Conventional Convex Probe: A Volunteer Study

**DOI:** 10.3390/diagnostics14090886

**Published:** 2024-04-24

**Authors:** Olivia Hänni, Lisa Ruby, Catherine Paverd, Thomas Frauenfelder, Marga B. Rominger, Alexander Martin

**Affiliations:** 1 Faculty of Medicine, University of Zurich, Dekanat Pestalozzistrasse 3, 8032 Zurich, Switzerland; 2 Institute of Diagnostic and Interventional Radiology, University Hospital Zurich, Rämistrasse 100, 8091 Zurich, Switzerland; 3 Department of Radiology, Memorial Sloan Kettering Cancer Center, 1275 York Avenue, New York, NY 10065, USAthomas.frauenfelder@usz.ch (T.F.);

**Keywords:** ultrasound, attenuation, fat, liver, fat quantification

## Abstract

The study aimed to investigate the feasibility of attenuation imaging (ATI) measurements using a linear probe on healthy volunteers and compare measurements with the conventional convex probe. Attenuation imaging measurements of the liver tissue were taken using ultrasound with a convex and a linear probe in 33 volunteers by two examining doctors, and the measurements were repeated 4–5 weeks later by one of them. The ATI values for the linear probe were in the range of the values for the convex probe for both examiners. Measurements did not change significantly for 32 out of 33 volunteers after 4–5 weeks when using the linear probe. The size of the region of interest (ROI) only impacted the ATI values for the convex probe; it did not affect the values taken with the linear probe. Healthy volunteers were measured, and their attenuation values were compared to those from a convex probe, commonly used in steatosis evaluation. When both probes were positioned in the same liver area, they showed good agreement in attenuation values, though depth significantly affected the measurements, with both probes providing different values at different depths. The study’s results aligned with previous research using the same system. Operator A and B’s results were compared, demonstrating similar ranges of values for both probes. The linear probe has been demonstrated to allow for superficial measurements and attain ATI values in line with that of the convex probe in the liver.

## 1. Introduction

Attenuation imaging is a useful clinical metric of the structure and disease processes throughout the body. Attenuation imaging measures the decrease in signal with depth caused by the interaction of imaging methods with tissue properties. Absolute attenuation values or relative values over time can provide significant helpful information about anatomical structure and disease progression. Attenuation imaging is currently performed using both MRI and ultrasound, and due to its usefulness, significant efforts have been made to develop the technology in recent years. This is especially true in the development of attenuation imaging in ultrasound due to the modality’s low cost and portability and the non-invasive and painless nature of the procedure [1,2,3,4]. Furthermore, no additional equipment is needed for examinations with ATI [5].

One beneficial area of attenuation imaging in ultrasound is in liver assessment. Hepatic steatosis is a condition present in 20–30% of the general population in the Western world [6,7], and non-alcoholic fatty liver disease (NAFLD) is now the most prevalent cause of chronic liver disease worldwide, ranging from simple steatosis to non-alcoholic steatohepatitis (NASH), fibrosis, cirrhosis, and hepatocellular carcinoma [6,8]. Hepatic steatosis is linked to various health complications, such as metabolic syndrome and cardiovascular disease. Steatosis can be improved by early treatment of the patient, and as such, methods for screening for steatosis are becoming ever more critical. Early detection and staging of steatosis can enable early intervention and behavioural changes in patients, thus leading, in some cases, to preventing disease progression into NASH and, ultimately, improving patient outcomes.

The current gold standard for quantifying steatosis is biopsy; however, sampling areas can provide unreliable results, and the invasive nature of a biopsy can lead to increased costs and potential medical complications. Therefore, there has been a significant effort in investigating and developing non-invasive alternatives for steatosis evaluation, specifically in MRI and ultrasound. Several studies have shown the potential of ultrasound attenuation index to detect hepatic steatosis [9,10,11]. Using a clinically approved linear probe, the same technique has yet to be fully explored. Canon has used attenuation imaging in the Aplio series of devices to determine the attenuation coefficient in assessing non-alcoholic fatty liver disease [11,12,13].

Measuring the attenuation coefficient with a linear probe might not only enable a better detection of superficial liver areas but also open new imaging opportunities in superficially located body areas, which were difficult to assess with the convex probe, such as breast tissue [14], muscles [15], or the thyroid [16].

No clinically approved linear array has had attenuation imaging available, and previous work with a clinically approved array has only been performed in phantoms [17]. An investigation of the performance of a clinically approved linear array and comparison to attenuation imaging on a convex array has not yet been performed. However, it would provide significant insight into using a linear array in in vivo attenuation imaging.

In order to assess the performance of ultrasound attenuation imaging on a linear array, this study compares attenuation measurements with a convex and linear probe in healthy volunteers on the liver, a well-known attenuation imaging target organ. Previous studies using the convex probe showed that ATI values correlate with steatosis of the liver [2,18,19]. Steatosis is graded using a four-grade system: S0, when there is no steatosis present; S1, mild, when less than 10% of hepatocytes are affected; S2, moderate, with between 10–30% of hepatocytes affected; and S3, severe, where greater than 30% of hepatocytes are impacted. 

This work provides the basis for understanding the behaviour and performance of attenuation on a linear array in humans, with an investigation into confounders such as ROI size and depth. This is with a view to progressing the technique for use on other, more superficial structures.

## 2. Materials and Methods

Approval for this prospective single-institutional study was obtained from both the institutional review board and local ethics committee (Kantonale Ethikkommission Zürich; KEK-ZH Nr. 2015-0323). Each volunteer for the study provided written informed consent prior to study participation and data collection. Consent for participation, as well as scientific evaluation and publication, was obtained for all volunteers. The volunteer data presented in this paper have not been published in previous studies. The study measurements were performed by two examiners on 33 volunteers. There were two examination dates for each volunteer with 4–5 weeks in between. ATI measurements of the liver were taken with a convex and a linear probe.

Thirty-three study participants were selected through local advertisements. Inclusion criteria for recruiting adult healthy volunteers in the study. The study was comprised of volunteers aged between 18 and 65 years old, with no history of liver or biliary disease or diabetes, a BMI from 17 to 30 kg/m^2^, no medication that may affect liver function for three months, and no history of surgery, trauma, or interventional procedure in the liver. Sex, age, BMI, and excessive alcohol use (above 14 units of alcohol per week) were assessed for all volunteers. The measurements were taken with volunteers having fasted for a minimum of 3 h.

The characteristics of volunteers can be found in Table 1. The volunteers comprised 19 females and 14 males, totalling 33 volunteers.

All ultrasound measurements in this study were performed on a TUS-AI800 (Aplio i800, Canon Medical System Corporation, Otawara, Japan) ultrasound scanner equipped using the i8CX1 convex array (centre frequency 4 MHz) and i11LX3 linear array (centre frequency 7 MHz). Two measurement sessions took place 4–5 weeks apart in September and October 2022 to show repeatability. All measurements from the first session were performed by two examiners, a radiology resident and an internal medicine resident. The internal medicine resident performed the second session. The volunteers were asked to lie supine with their right arm behind their head. The examination took place in a secluded compartment. Volunteers were draped with towels during scanning for privacy. Commercially available ultrasound gel (UL-01, Skintact, Healthcare, London, UK) was applied on the respective probe, which was then placed in the intercostal window on the midclavicular or anterior axillary line.

### 2.1. Measurement Protocol

**Figure 1:**
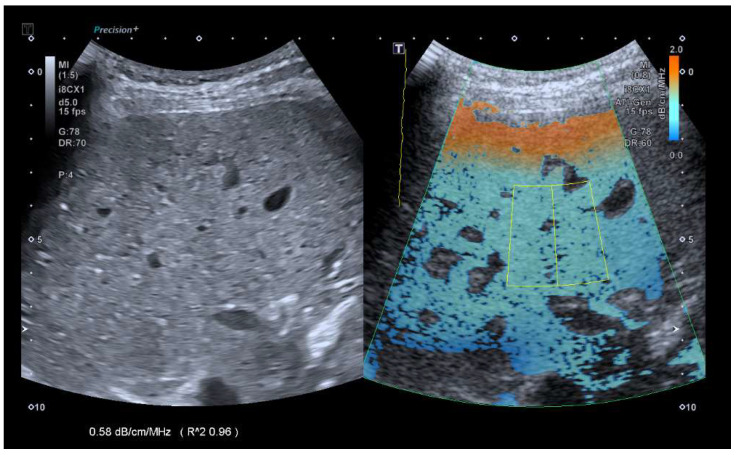
Exemplary images of b-mode and ATI measurements for both the convex probe.

The ATI mode was selected with dual screen and a depth of 10 cm. The area of interest (AOI) box was enlarged to its maximum size and placed in the middle of the frame. The volunteers were asked to inhale and slowly exhale, and an image was obtained and frozen in segment VI/VII. A region of interest (ROI) was then placed in an area 1 cm below the liver capsule with an R2 > 0.9 (Figure 1). Four additional repeated measurements were then obtained by removing the probe and placing it in the same area.

**Figure 2:**
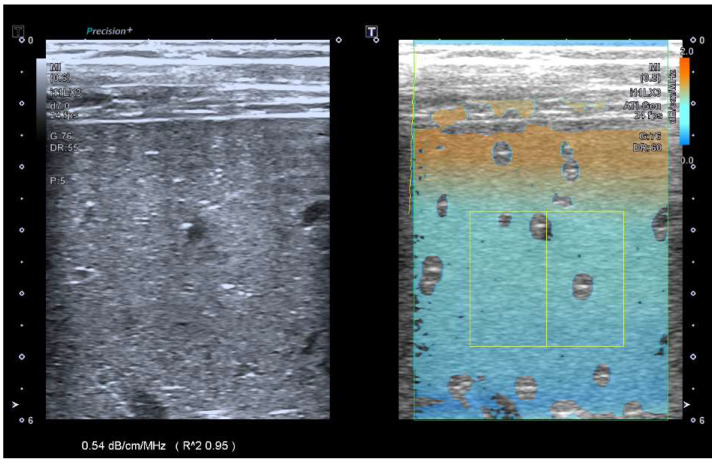
Example image of linear probe ATI imaging with b-mode image on the left and ATI overlay on the right.

The pre-set ATI setting for general imaging (“ATI-Gen”) was selected, characterized by a dual screen and a depth of 6 cm. The area of interest (AOI) box was enlarged to its maximum size and placed in the middle of the frame. The volunteers were asked to inhale and slowly exhale, and an image was obtained and frozen in segment VI/VII. A region of interest (ROI) (4 × 2 cm) was then placed in an area 1 cm below the liver capsule with an R2 > 0.9 (Figure 2). An additional four repeated measurements were obtained.

### 2.2. Statistics

Statistical analysis was performed using GraphPad Prism 9.5.1. Unpaired *t*-tests were conducted to compare the measurement values for each volunteer and each method or timeframe. A *p*-value of less than 0.05 was considered statistically significant.

Intraobserver variability is presented within this study as a percentage and measured using the individual standard deviations for each pair of measurements, week 0 and week 4, using the following formula.

√[(M1 − M2)^2^/2]/[(M1 + M2)/2] (1)

where ‘M1’ is measurements in week 0, ‘M2’ is in week 4.

## 3. Results

### 3.1. Convex versus Linear Probe

A comparison of the convex probe and linear probe measurements were initially made for each Operator and are presented in Figure 3, Figure 4, Figure 5, Figure 6, Figure 7, Figure 8, Figure 9, Figure 10, Figure 11, Figure 12, Figure 13, Figure 14, Figure 15 and Figure 16.

The range of average values obtained by Operator A were from 0.50 to 0.89 dB/cm/MHz using the convex probe. Of the 33 volunteers for this study, 15 of those measured fell under the lowest attenuation values used to grade steatosis. The range used to determine S1 is between 0.59–0.69 dB/cm/MHz. The remaining 18 volunteers fall within the literature values used to classify S1. 28 of the volunteers have attenuation values less than 0.65 dB/cm/MHz, used as the lower cutoff for S2 grading.

**Figure 3:**
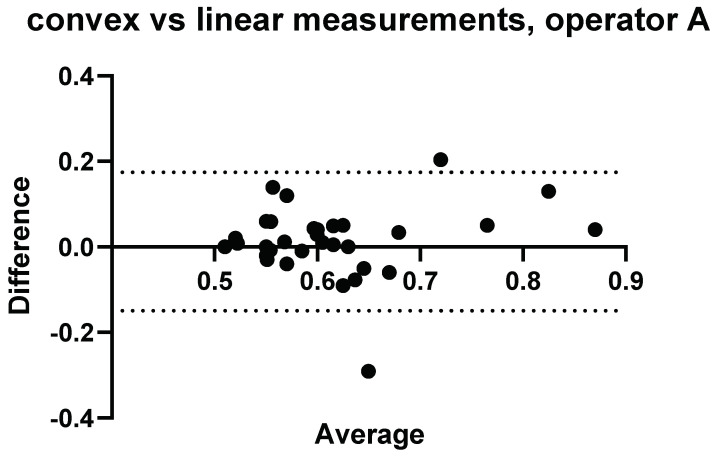
Operator A, comparison of convex probe measurements versus linear probe measurements in volunteers. Results here represent measurements taken at week 0.

**Figure 4:**
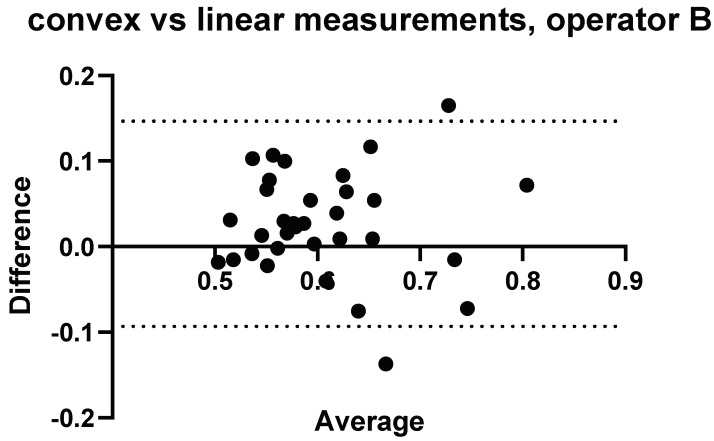
Operator B, comparison of convex probe measurements versus linear probe measurements in volunteers. Results here represent measurements taken at week 0. A significant difference in measurements is seen for two volunteers.

The results for Operator A ranged from 0.49 to 0.85 dB/cm/MHz using the linear probe. Of the 33 volunteers, 14 fell under the 0.59 dB/cm/MHz cutoff. Ten volunteers fell within the range of 0.59–0.69 dB/cm/MHz, and five volunteers fell within the range of 0.65–0.72 dB/cm/MHz, used to classify potential S2 classification, with four volunteers having values greater than 0.72 dB/cm/MHz. Operator B recorded values in the 0.49–0.78 dB/cm/MHz range. Nineteen of the volunteers showed values < 0.59 dB/cm/MHz, with 10 volunteers falling within the 0.59–0.69 dB/cm/MHz range. There were four volunteers with values greater than 0.72 dB/cm/MHz. There is a statistical difference between four of the measurements as shown on Figure 3. A comparison for measurements using convex and linear probes can be seen for each operator in Figure 3 and Figure 4 respectively. 

### 3.2. Intraobserver Comparisons

**Figure 5:**
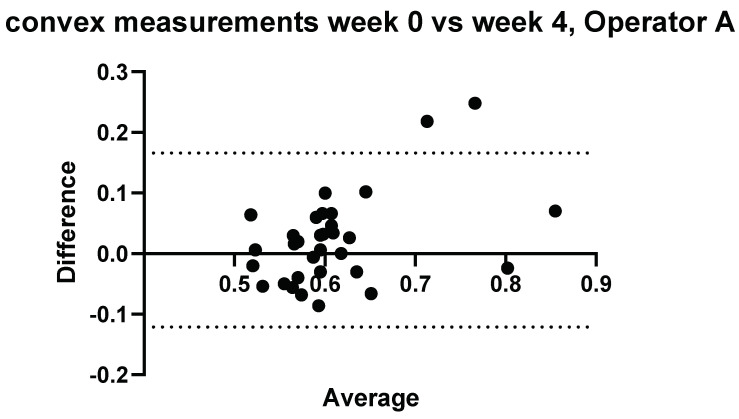
Intra-operator: Comparison of Operator A measurements at week 0 versus Operator A measurements at week 4–5. Comparison of convex probe measurements, with a significant difference for volunteers 2, 3, and 17.

**Figure 6:**
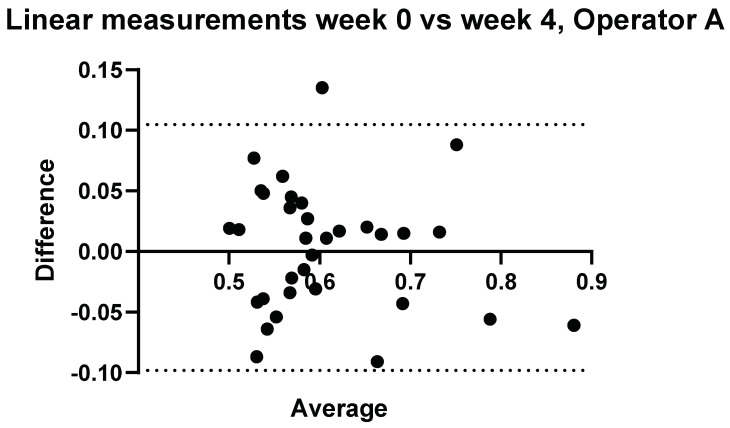
Intra-operator: comparison of Operator A measurements at week 0 versus Operator A measurements at week 4–5. Comparison of linear probe measurements, with one significant difference in measurement values for volunteer 29.

Further to Operator A and Operator B’s measurements, measurements were conducted again on the same volunteers 4 weeks later by Operator A. The results comparing measurements performed by Operator A at week 0 and week 4 can be seen in Figure 5 and Figure 6.

Three measurements showed a significant difference between measurements taken at week 0 and week 4 using the convex probe.

One measurement showed a significant difference between measurements taken at week 0 and week 4 using the linear probe.

#### Relative Intraobserver Variability

Measurements conducted by Operator A at week 0 and repeat measurements at week 4 are presented below in Table 2 for convex measurements and Table 3 for linear measurements. There was an average variance of 6.1% between week 0 and week 4 when using the convex probe and 5.0% when using the linear.

### 3.3. Interobserver Comparisons

Operator B obtained values ranging from 0.49 to 0.84 dB/cm/MHz with the convex probe. There were 14 volunteers measured with values less than 0.59 dB/cm/MHz. Of these 14, 11 matched operator A’s results. The volunteers whose results did not fall within the same category differed by a maximum of 10% of recorded values. Of the remaining 19 volunteers, 14 fall within the 0.59–0.69 dB/cm/MHz range for S1 stagging, with 6 volunteers having attenuation values between 0.65 and 0.72 dB/cm/MHz. Finally, there were three volunteers with values greater than 0.72 dB/cm/MHz.

Operator B recorded values in the 0.49–0.78 dB/cm/MHz range. Nineteen of the volunteers with values < 0.59 dB/cm/MHz, with ten volunteers falling within the 0.59–0.69 dB/cm/MHz range. There were four volunteers with values greater than 0.72 dB/cm/MHz. 

**Figure 7:**
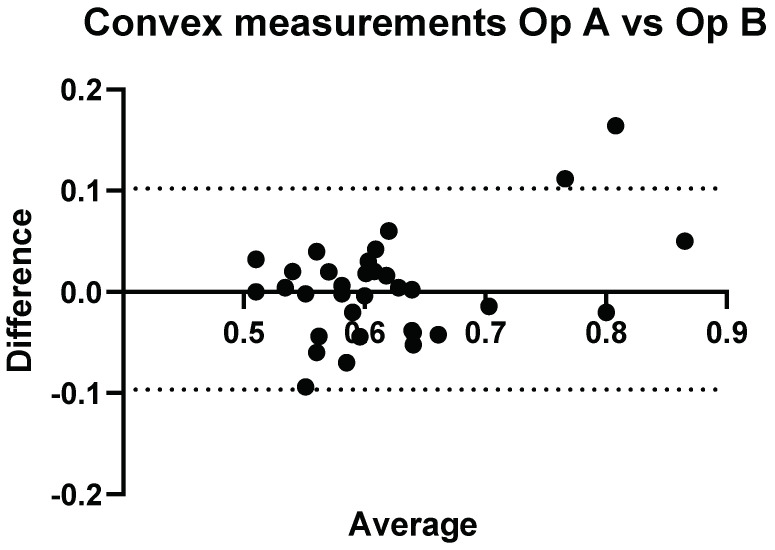
Inter-operator: comparison of Operator A versus Operator B. Comparison of Convex probe measurements. Results here represent measurements taken at week 0, with a significant difference for volunteers 14, 26, and 32.

**Figure 8:**
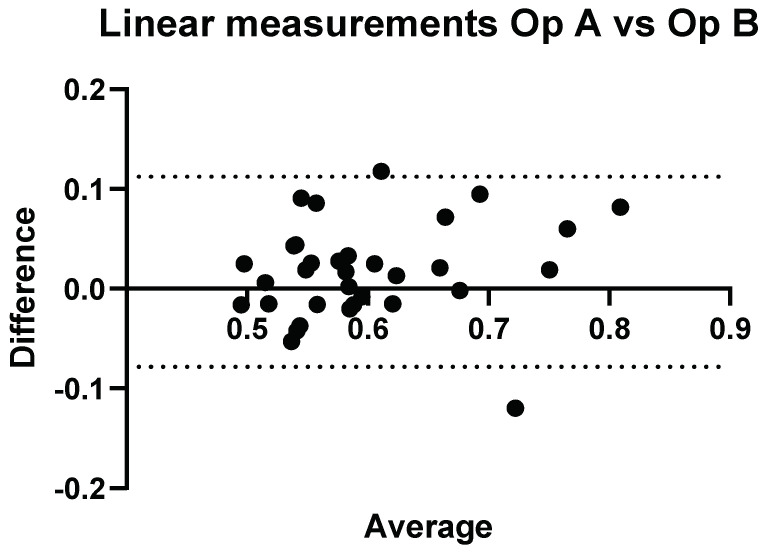
Inter-operator: comparison of Operator A versus Operator B. Comparison of linear probe measurements. Results here represent measurements taken at week 0, with a significant difference for volunteers 4 and 29.

A direct comparison of Operator A and B’s convex and linear measurements can be seen in Figure 7 and Figure 8, respectively.

Three of the volunteers had statistically significant different measurement values when comparing Operator A and B’s measurements, with *p* < 0.05.

Again, two of the volunteers had significant differences in their measurement values. The range of values for Operator A and Operator B were 0.49–0.85 dB/cm/MHz and 0.49–0.78 dB/cm/MHz.

### 3.4. Confounders: ROI-Size and Insertion Depth

**Figure 9:**
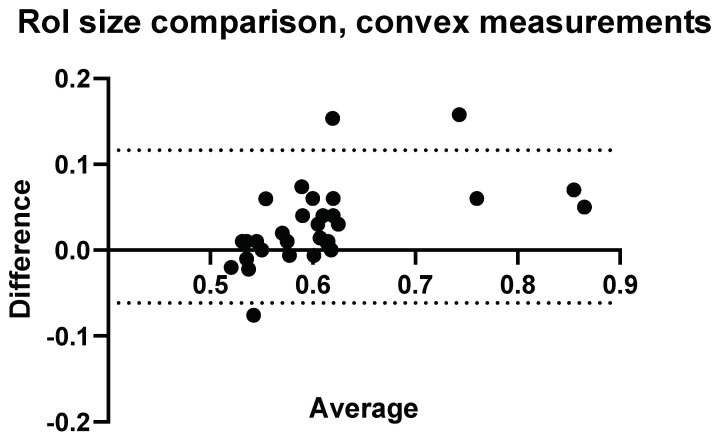
ROI size: comparison of Operator A measurements at week 0 with changing ROI size. Comparison of convex probe measurements at 1 cm below the liver capsule.

**Figure 10:**
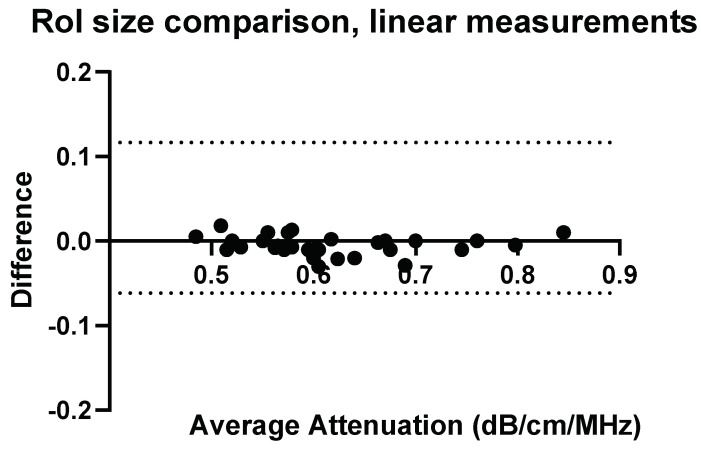
ROI size: comparison of Operator A measurements at week 0 with changing ROI size, 2 × 2 cm ROI vs. 2 × 4 cm (h × w). Comparison of linear probe measurements at 1 cm below the liver capsule.

**Figure 11:**
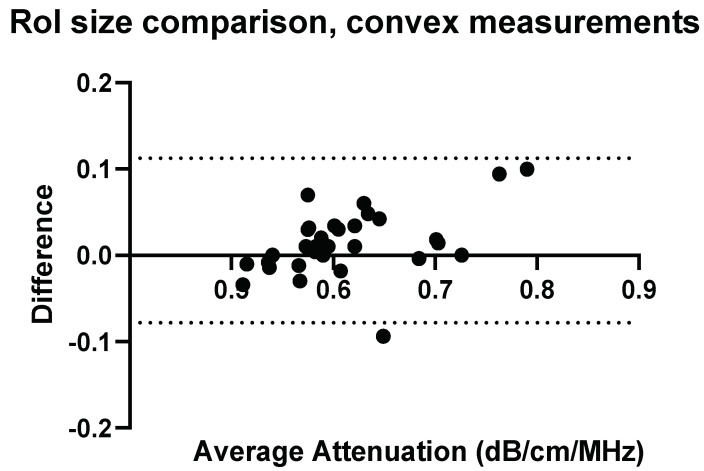
ROI size: comparison of Operator B measurements with changing ROI size. Comparison of convex probe measurements at 1 cm below the liver capsule.

**Figure 12:**
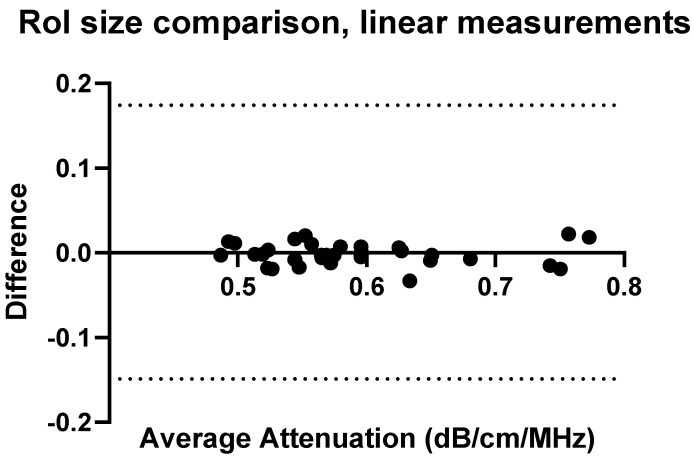
ROI size: comparison of Operator B measurements with changing ROI size, 2 × 2 cm ROI vs. 2 × 4 cm (h × w). Comparison of linear probe measurements at 1 cm below the liver capsule. No significant differences.

To fully understand the impact of potential confounders, the effects of region of interest size and depth on measurement values were examined for Operators A and B. The effects of region of interest size can be seen in Figure 9, Figure 10, Figure 11 and Figure 12.

With a change in the measurement area of the region of interest, there was a significant effect on five of the measurement values using the convex probe. There was no significant difference when changing the area of the region of interest using the linear probe. 

With a change in the measurement area of the region of interest, there was a significant difference for five of the measurement values using the convex probe for Operator B. Again, there was no significant difference when changing the area of the region of interest when using the linear probe with Operator B.

**Figure 13:**
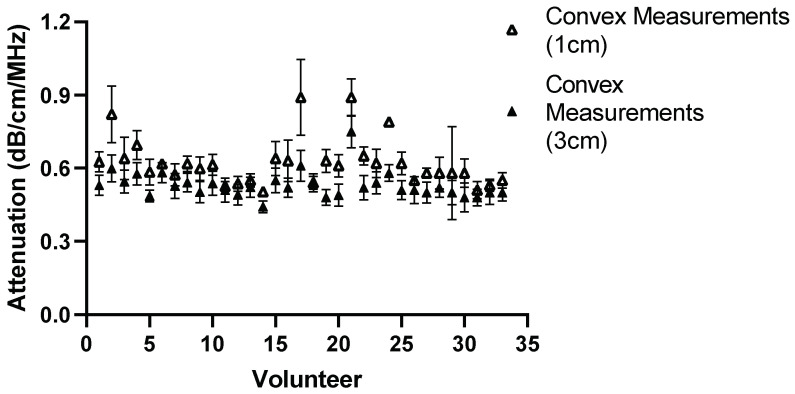
Comparison of Operator A measurements at week 0 with changing position of ROI box placement. Comparison of convex probe measurements at 1 cm and 3 cm below liver capsule. Significant difference in measurement values for 21 of the 33 volunteers.

**Figure 14:**
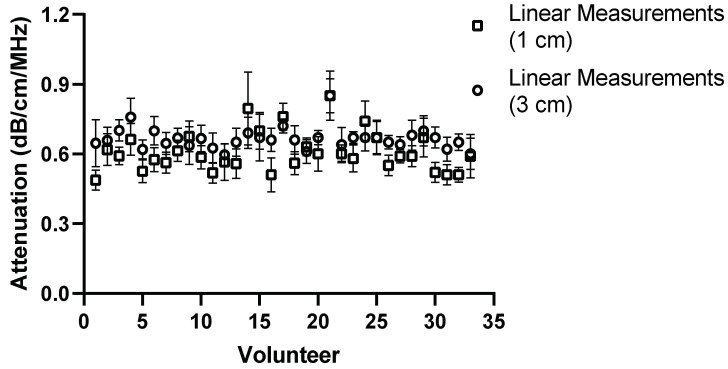
Comparison of Operator A measurements at week 0 with changing position of ROI box placement. Comparison of linear probe measurements at 1 cm and 3 cm below the liver capsule. Significant difference in 17 of the measurement values.

**Figure 15:**
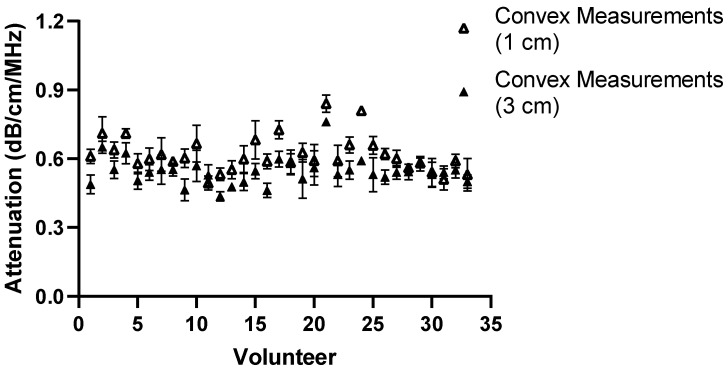
Comparison of Operator B measurements at week 0 with changing position of ROI box placement. Comparison of convex probe measurements at 1 cm and 3 cm below the liver capsule. Significant difference in values in 19 volunteers.

**Figure 16:**
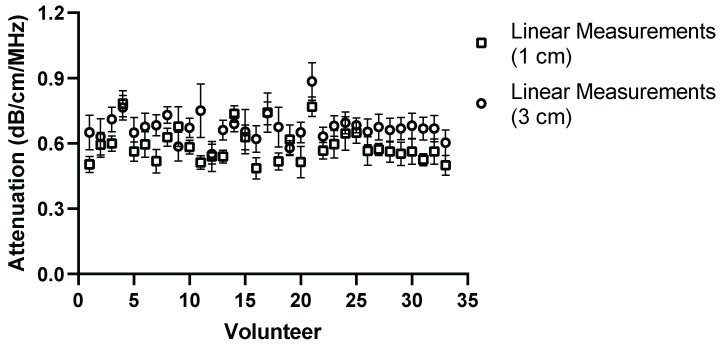
Comparison of Operator B measurements at week 0 with changing position of ROI box placement. Comparison of linear probe measurements at 1 cm and 3 cm below the liver capsule. Significant difference in values in 21 volunteers.

The effect of depth was also examined by moving the region of interest to deeper into the liver, the results of which can be seen in Figure 13, Figure 14, Figure 15 and Figure 16 for both Operator A and B.

The same comparison for Operator B can be seen below in Figure 15 and Figure 16.

There was a significant difference in measurement values for 19 of the volunteers for Operator B. The same general trend in the measurements emerged that the attenuation value tends to be lower when using the convex probe at a deeper position. The results of Operator B’s linear measurements also show a significant difference in values for 21 volunteers. Again, like Operator A, a general trend appeared; the attenuation values tend to increase as the ROI is placed deeper.

## 4. Discussion

The results show the feasibility of ATI measurements using a linear probe in healthy volunteers. The measured values are within the range of the convex probe. The convex probe has been extensively researched, with many studies evaluating the measurements obtained using a convex probe and either MRI measurements or histological confirmation of steatosis. 

For the purposes of the work presented here, when the linear and convex probes are positioned in the same area of the liver, they are in good agreement with measured attenuation values. This is, however, significantly affected by depth. The convex and linear probes return significantly different values based on the measurement depth, as shown in Figure 13, Figure 14, Figure 15 and Figure 16. 

The range of values presented in this study is similar to those presented in other studies examining the ability of the convex probe using the same system (Canon, Aplio i800) as used here. In terms of grading, the wide range of attenuation values and overlapping of some of these values is unsurprising. As confirmed by histology, the grading of S0 being <5%, S1 between 5–33%, S2 between 33–66%, and S3 > 66% of hepatocytes containing fat [20], the distribution of where these cells exist in the liver can never be fully known. However, imagining modalities offer the opportunity to explore multiple areas of the liver in one setting and offer a low-cost, non-invasive alternative to biopsy. 

The results obtained for both Operator A and Operator B were used to compare the ability of the linear probe to gather ATI values within the liver. 

The use of the same cutoffs when using a convex probe and a linear probe must be noted here. There are currently no cutoff ranges available for the linear probe to confirm steatosis, and those used here are only to show how the measurements compare with the convex probe. However, in future, if the linear array is used in the assessment of, for example, superficial liver lesions, such cutoffs would need to be further investigated and developed.

Comparing Operator A’s results for the convex and linear probe, the range of values recorded were 0.50–0.89 dB/cm/MHz for the convex probe and 0.49–0.85 dB/cm/MHz for the linear. Grouping the volunteers into no steatosis (<0.59 dB/cm/MHz), S1 (0.59–0.69), or S2 (0.65–0.72), and comparing between the values recorded with each probe, results in 15 volunteers with no indication of steatosis when measured using the convex versus 18 volunteers using the linear probe. For ‘S1’, there were 13 volunteers within the range of 0.59–0.69 dB/cm/MHz; with the linear probe, there were 10 volunteers. For ‘S2’, there were six volunteers who fell between 0.65–0.72 dB/cm/MHz and nine when using the linear probe. 

Operator B recorded values within the range of 0.49–0.84 dB/cm/MHz using the convex probe and values of 0.49–0.78 dB/cm/MHz using the linear probe. The volunteers fell into the following categories: 14 had values less than 0.59 dB/cm/MHz using the convex probe, compared with 19 using the linear probe. Using the convex measured values, 14 fell within 0.59–0.69 dB/cm/MHz, with 10 in the same range using the linear. Finally, there were three volunteers recorded with >0.72 dB/cm/MHz values using the convex probe, and four using the linear. 

While the results for Operators A and B share a similar range whether using the convex probe or the linear probe, the inter-operator measurements show significant differences for four volunteers when using the convex probe and three when using the linear probe.

The effect of region of interest was also investigated for both the convex and linear probes. For operators A and B, changing the region of interest size in the X direction (Figure 9, Figure 10, Figure 11 and Figure 12) and changing the measurement area significantly affected the measurements taken with the convex probe. One explanation for this is the size difference in the smallest and larger areas being measured with the change in ROI window size. The liver is highly vascularised and images containing a vessel segment can significantly impact on the US machine and the output of attenuation values. Comparatively, the size of the region of interest for the linear probe evaluation had no significant effect on either operator A or B’s measurements. However, the imaging window is much smaller while using the linear probe. This would indicate that when establishing a protocol, or moving forward in a patient pilot study, region of interest size can be considered less important than other confounders discussed here. 

Depth played a significant role for operators A and B while using the convex and linear probes. This is unsurprising given the results also seen when measuring attenuation using a linear probe in phantoms [17]. There was a significant difference in measurement values for 21 volunteers obtained by moving the ROI deeper. A general trend in the measurements can also be seen in that, at a deeper position, the attenuation value tends to be lower when using the convex probe. 

The results of Operator A’s linear measurements also show a significant difference in values for 17 of the volunteers. There is also a general trend, however: as the ROI is placed deeper, the attenuation values tend to increase. 

A change in depth of 2 cm for the positioning of the region of interest showed a significant difference in the values subsequently recorded. For the convex probe, there was a significant difference in measurements for 21 and 19 volunteers for Operators A and B. For the linear, measurements were significantly impacted for 17 and 21 of the volunteers for each Operator, with an average increase of 0.081 dB/cm/MHz for each Operator at the increased depth. 

The overall decrease in the attenuation coefficient can be seen both in individual measurements and examining the overall group results for convex measurements. The measurements at 1 cm for the convex probe have a range of attenuation coefficient values of 0.49–0.84 dB/cm/MHz, whereas at 3 cm, this range decreases to 0.44–0.76 dB/cm/MHz. These results are in agreement with those found by Ferraioli et al. [21]. There is a clear depth dependency when measuring with the convex probe in the liver, with an average decrease of 0.074 dB/cm/MHz in measurement positions. Ferrailoli et al. show that depth has a significant effect on ATI values, with as little of a change as 0.5–1 cm in positioning of the measurement box causing a change of 0.05 dB/cm/MHz [21]. One explanation for this is the reverberations that can be caused by the US beam passing through the liver capsule. The establishment of a robust protocol for measurement position and depth is critical for the accurate translation of results into clinical practice.

Two trends also appeared as the box was placed deeper. With the convex probe, the trend was for the attenuation values to decrease, which in part can be explained by avoiding reverberations. However, with the linear probe, the opposite trend is exhibited. The probe itself can, in turn, explain this. The imaging depth with a linear probe is limited, and by extending the measurement area, the limits of the probe are being exposed and thus the attenuation values deeper in the liver appear to increase. 

To find depth to be a confounder is not surprising given the difficulties with depth when imaging and using other techniques such as shear wave elastography with ultrasound analysis. While shear wave elastography is used to study fibrosis and the purpose of attenuation imaging is steatosis, there are obvious parallels in considerations that must be made by clinicians, including being aware of how the depth of measurement may impact the quality and reliability of the measurement value. Measurements such as shear wave elastography and velocity have been demonstrated to be influenced by depth of measurement [22,23,24,25,26], as attenuation imaging is also now being shown to be. 

The study presented here demonstrated that measurements are consistent between different operators following the same protocol, demonstrating excellent repeatability. While the number of volunteers available for this pilot study was limited, no drop-out or values had to be excluded as part of the analysis. However, there are limitations when it comes to the available volunteers as they were all healthy, with no indication of liver disease. 

The linear probe, when used for B-mode imaging, is limited as to which depth it can scan, and as such, for volunteers with thicker muscle or fat layers, the results can become less consistent, and the placement of the ROI can be at the very limit of the capabilities of the linear probe. This is a significant consideration for the potential use of the linear probe to measure ATI in the liver of those suspected of having steatosis due to the nature of the disease and the common effects on patients with steatosis. High attenuation of ultrasound signals leads to poor image quality at depth, especially when evaluating deep abdominal organs. Ultrasound signal attenuation depends on the tissue’s physical properties and depth of the measurement [27]. For example, when imaging a fatty liver, there is an associated increase in ultrasound signal attenuation due to increased backscatter [13]. This increase in attenuation causes a decrease in signal intensity, leading to poor image quality in deeper regions.

The next step for additional comparison will include volunteers with known pathologies or liver disease to assess the clinical application and allow for additional protocol development. As part of future studies, further investigation into depth and its impact upon linear probe ATI measurements is required to understand the full impact for patients, as it is seen that with ‘healthy’ volunteers, measurements are more difficult with additional fat or muscle layers. 

There is also the opportunity to explore more superficial tissue, such as breast tissue, and to investigate pathologies such as sarcopenia.

## 5. Conclusions

The results of the study show that ATI values attainable using the convex probe are also attainable using a linear probe. However, several important considerations are needed to achieve this, including the limitation and effects of depth on measurements when using the linear probe. The linear probe, while obtaining similar values to that of the convex probe at 1 cm below the liver capsule, requires additional testing in patients who are known to have steatosis to establish whether this effect of depth will mean an effective protocol cannot be established for steatosis classification. The linear probe is known to have this challenge in all forms of ultrasound imaging, and therefore, it is not surprising when using attenuation imaging; however, by demonstrating the functionality of the probe at clinical depths, it opens other avenues of exploration, such as paediatric liver measurements and thyroid, musculoskeletal, and breast tissue measurements, all of which provide a more superficial target for assessment, where the linear probe is in its element. The ability of the probe to measure attenuation at a limited depth while limiting liver measurements may allow for further investigation into more superficial tissue, such as muscle or breast tissue. Further investigation into these areas to confirm is required.

## Figures and Tables

**Table 1:** Volunteer characteristics, *n* = 33. BMI—Body Mass Index. The range of group characteristics is presented in brackets, with average.

	*n* (Range)
Age (years)	34.52 (24.5–63.3)
Sex	19 (f)/14 (m)
Weight (kg)	67.3 (52–86)
Height (m)	1.72 (1.53–1.90)
BMI (kg/m^2^)	22.6 (18.0–28.0)
Alcohol > 14 units/week	3 yes/30 no

**Table 2:** Relative intraobserver variability for Operator A and convex measurements.

Volunteer	Convex Week 0 dB/cm/MHz	Convex Week 4 dB/cm/MHz	Relative Intraobserver Variability
1	0.63	0.59	3.9%
2	0.82	0.60	21.6%
3	0.64	0.57	7.7%
4	0.70	0.59	11.2%
5	0.58	0.59	0.7%
6	0.62	0.62	0.0%
7	0.57	0.56	2.0%
8	0.62	0.68	7.2%
9	0.60	0.59	0.7%
10	0.61	0.58	3.8%
11	0.53	0.52	0.8%
12	0.54	0.59	7.0%
13	0.55	0.64	10.3%
14	0.50	0.56	7.2%
15	0.64	0.61	2.9%
16	0.63	0.58	5.4%
17	0.89	0.64	22.9%
18	0.54	0.61	8.4%
19	0.63	0.56	7.8%
20	0.61	0.58	3.6%
21	0.89	0.82	5.8%
22	0.65	0.55	11.8%
23	0.62	0.56	7.2%
24	0.79	0.81	2.1%
25	0.62	0.65	3.3%
26	0.55	0.59	5.0%
27	0.58	0.61	3.6%
28	0.58	0.56	2.5%
29	0.58	0.55	3.8%
30	0.58	0.55	3.8%
31	0.51	0.53	2.7%
32	0.53	0.58	6.4%
33	0.55	0.49	8.7%

**Table 3:** Relative intraobserver variability for Operator A and linear measurements.

Volunteer	Linear Week 0 dB/cm/MHz	Linear Week 4 dB/cm/MHz	Relative Intraobserver Variability
1	0.49	0.57	11.6%
2	0.62	0.71	9.7%
3	0.59	0.55	5.6%
4	0.66	0.64	2.2%
5	0.53	0.58	6.9%
6	0.58	0.59	1.8%
7	0.56	0.51	6.3%
8	0.61	0.60	1.3%
9	0.68	0.66	1.5%
10	0.59	0.55	4.5%
11	0.52	0.56	5.1%
12	0.57	0.49	10.3%
13	0.56	0.58	2.7%
14	0.80	0.71	8.3%
15	0.70	0.69	1.5%
16	0.51	0.57	8.3%
17	0.76	0.82	5.0%
18	0.56	0.51	6.6%
19	0.63	0.61	1.9%
20	0.60	0.57	3.3%
21	0.85	0.91	4.9%
22	0.60	0.56	4.9%
23	0.58	0.61	3.7%
24	0.74	0.72	1.5%
25	0.67	0.71	4.4%
26	0.55	0.58	4.2%
27	0.59	0.59	0.4%
28	0.59	0.58	1.3%
29	0.67	0.54	15.8%
30	0.52	0.50	2.5%
31	0.51	0.49	2.7%
32	0.51	0.55	5.6%
33	0.59	0.53	7.8%

## Data Availability

The data of this study is available on request.

## References

[1] Yang K.C., Liao Y.Y., Tsui P.H., Yeh C.K. (2019). Ultrasound imaging in nonalcoholic liver disease: Current applications and future developments. Quant. Imaging Med. Surg..

[2] Bae J.S., Lee D.H., Lee J.Y., Kim H., Yu S.J., Lee J.-H., Cho E.J., Lee Y.B., Han J.K., Choi B.I. (2019). Assessment of hepatic steatosis by using attenuation imaging: A quantitative, easy-to-perform ultrasound technique. Eur. Radiol..

[3] Ozturk A., Grajo J.R., Gee M.S., Benjamin A., Zubajlo R.E., Thomenius K.E., Anthony B.W., Samir A.E., Dhyani M. (2018). Quantitative Hepatic Fat Quantification in Non-alcoholic Fatty Liver Disease Using Ultrasound-Based Techniques: A Review of Literature and Their Diagnostic Performance. Ultrasound Med. Biol..

[4] Liao Y.Y., Yang K.C., Lee M.J., Huang K.C., Chen J.D., Yeh C.K. (2016). Multifeature analysis of an ultrasound quantitative diagnostic index for classifying nonalcoholic fatty liver disease. Sci. Rep..

[5] Hsu P.K., Wu L.S., Su W.W., Su P.Y., Chen Y.Y., Hsu Y.C., Yen H.H., Wu C.L. (2021). Comparing the controlled attenuation parameter using FibroScan and attenuation imaging with ultrasound as a novel measurement for liver steatosis. PLoS ONE.

[6] Nassir F., Rector R.S., Hammoud G.M., Ibdah J.A. (2015). Pathogenesis and Prevention of Hepatic Steatosis. Gastroenterol. Hepatol..

[7] Mehta S.R., Thomas E.L., Bell J.D., Johnston D.G., Taylor-Robinson S.D. (2008). Non-invasive means of measuring hepatic fat content. World J. Gastroenterol..

[8] Byrne C.D., Targher G. (2015). NAFLD: A multisystem disease. J. Hepatol..

[9] Gatos I., Drazinos P., Yarmenitis S., Theotokas I., Koskinas J., Koullias E., Mitranou A., Manesis E., Zoumpoulis P.S. (2022). Liver Ultrasound Attenuation: An Ultrasound Attenuation Index for Liver Steatosis Assessment. Ultrasound Q..

[10] Alves V.P.V., Dillman J.R., Tkach J.A., Bennett P.S., Xanthakos S.A., Trout A.T. (2022). Comparison of Quantitative Liver US and MRI in Patients with Liver Disease. Radiology.

[11] Ferraioli G., Maiocchi L., Savietto G., Tinelli C., Nichetti M., Rondanelli M., Calliada F., Preda L., Filice C. (2021). Performance of the Attenuation Imaging Technology in the Detection of Liver Steatosis. J. Ultrasound Med..

[12] Ferraioli G., Maiocchi L., Raciti M.V., Tinelli C., De Silvestri A., Nichetti M., De Cata P., Rondanelli M., Chiovato L., Calliada F. (2019). Detection of Liver Steatosis with a Novel Ultrasound-Based Technique: A Pilot Study Using MRI-Derived Proton Density Fat Fraction as the Gold Standard. Clin. Transl. Gastroenterol..

[13] Jesper D., Klett D., Schellhaas B., Pfeifer L., Leppkes M., Waldner M., Neurath M.F., Strobel D. (2020). Ultrasound-Based Attenuation Imaging for the Non-Invasive Quantification of Liver Fat—A Pilot Study on Feasibility and Inter-Observer Variability. IEEE J. Transl. Eng. Heal Med..

[14] Steyaert L. (2000). Doppler sonography in breast pathology. JBR-BTR.

[15] Balius R., Pedret C., Iriarte I., Sáiz R., Cerezal L. (2019). Sonographic landmarks in hamstring muscles. Skelet. Radiol..

[16] Hegedüs L. (2001). Thyroid ultrasound. Endocrinol. Metab. Clin. N. Am..

[17] Hänni O., Ruby L., Paverd C., Frauenfelder T., Rominger M.B., Martin A. (2024). Confounders of Ultrasound Attenuation Imaging in a Linear Probe Using the Canon Aplio i800 System: A Phantom Study. Diagnostics.

[18] Sasso M., Beaugrand M., De Ledinghen V., Douvin C., Marcellin P., Poupon R., Sandrin L., Miette V. (2010). Controlled attenuation parameter (CAP): A novel VCTE^TM^ guided ultrasonic attenuation measurement for the evaluation of hepatic steatosis: Preliminary study and validation in a cohort of patients with chronic liver disease from various causes. Ultrasound Med. Biol..

[19] Tada T., Iijima H., Kobayashi N., Yoshida M., Nishimura T., Kumada T., Kondo R., Yano H., Kage M., Nakano C. (2019). Usefulness of Attenuation Imaging with an Ultrasound Scanner for the Evaluation of Hepatic Steatosis. Ultrasound Med. Biol..

[20] Kleiner D.E., Brunt E.M., Van Natta M., Behling C., Contos M.J., Cummings O.W., Ferrell L.D., Liu Y.-C., Torbenson M.S., Unalp-Arida A. (2005). Design and validation of a histological scoring system for nonalcoholic fatty liver disease. Hepatology.

[21] Ferraioli G., Raimondi A., Maiocchi L., De Silvestri A., Poma G., Kumar V., Barr R.G. (2023). Liver Fat Quantification With Ultrasound: Depth Dependence of Attenuation Coefficient. J. Ultrasound Med..

[22] Bruce M., Kolokythas O., Ferraioli G., Filice C., O’Donnell M. (2017). Limitations and artifacts in shear-wave elastography of the liver. Biomed. Eng. Lett..

[23] Rominger M.B., Kälin P., Mastalerz M., Martini K., Klingmüller V., Sanabria S., Frauenfelder T. (2018). Influencing Factors of 2D Shear Wave Elastography of the Muscle—An Ex Vivo Animal Study. Ultrasound Int. Open.

[24] Hwang J.A., Jeong W.K., Song K.D., Kang K.A., Lim H.K. (2019). 2-D Shear Wave Elastography for Focal Lesions in Liver Phantoms: Effects of Background Stiffness, Depth and Size of Focal Lesions on Stiffness Measurement. Ultrasound Med. Biol..

[25] Shin H.J., Kim M.J., Kim H.Y., Roh Y.H., Lee M.J. (2016). Comparison of shear wave velocities on ultrasound elastography between different machines, transducers, and acquisition depths: A phantom study. Eur. Radiol..

[26] Yamanaka N., Kaminuma C., Taketomi-Takahashi A., Tsushima Y. (2012). Reliable measurement by virtual touch tissue quantification with acoustic radiation force impulse imaging: Phantom study. J. Ultrasound Med..

[27] Taylor K.J., Riely C.A., Hammers L., Flax S., Weltin G., Garcia-Tsao G., Conn H.O., Kuc R., Barwick K.W. (1986). Quantitative US attenuation in normal liver and in patients with diffuse liver disease: Importance of fat. Radiology.

